# Supplementary material for: Spatially resolved quantification of oxygen consumption rate in ex vivo lymph node slices
Source: Analyst. 2024 Mar 27;149(9):2609–20. doi: 10.1039/d4an00028e (PMC11056769; doi:10.1039/d4an00028e)
Supplement: AN-149-D4AN00028E-s004 [file AN-149-D4AN00028E-s004.pdf]

Electronic Supplementary Material (ESI) for Analyst.
This journal is © The Royal Society of Chemistry 2024

Supplemental Information for

Spatially resolved quantification of oxygen consumption rate in ex vivo lymph node slices

Parastoo Anbaei*, Marissa G. Stevens[§ǀ], Alexander G. Ball[‡ǀ], Timothy N.J. Bullock[§ǀ], and Rebecca R. Pompano*[†ǀ]

*Department of Chemistry, University of Virginia College of Arts and Sciences, Charlottesville, Virginia 22904, United states

[†]Department of Biomedical Engineering, University of Virginia School of Engineering and Applied Sciences, Charlottesville, Virginia 22904, United States

[‡]Department of Microbiology Cancer Biology and Immunology, University of Virginia, Charlottesville, Virginia 22903, United States

[§]Department of Pathology, University of Virginia, Charlottesville, Virginia 22903, United States

[ǀ]Carter Immunology Center and UVA Cancer Center, University of Virginia, Charlottesville, Virginia 22903, United States

Contents

Supplemental Figures 2

Captions for supplemental movies 3

Supplemental Tables 4

Supplemental assumptions and calculations 4

Dissolved oxygen calculations and assumptions: Converting mmHg to mM O_2 4

Shear stress calculation for flow of PBS in perfusion chamber 5

Rates of oxygen consumption: Assumptions and calculations 6

Supporting references 8

Supplemental Figures

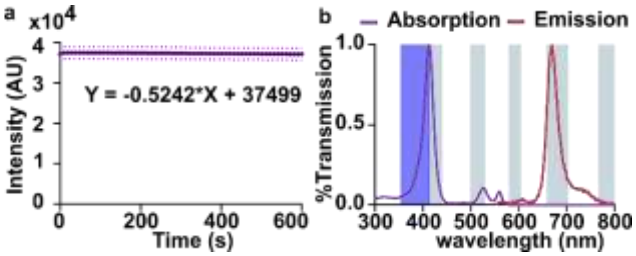

Figure S1: Characterization of the photostability and absorption and emission spectra of the PdTFPP sensor. (a) Luminescent intensity (arbitrary units) of the PdTFPP oxygen sensor film in gas phase 100% nitrogen over 45 min. Images were collected every 10 s at 150 ms exposure time; the shutter was closed between exposures. (b) Absorption (purple) and emission (red) spectra of PdTFPP. The wavelengths of the pentapass emission filter (grey; Zeiss Filter set LED 112) and of the DAPI excitation LED (dark blue) are also shown.

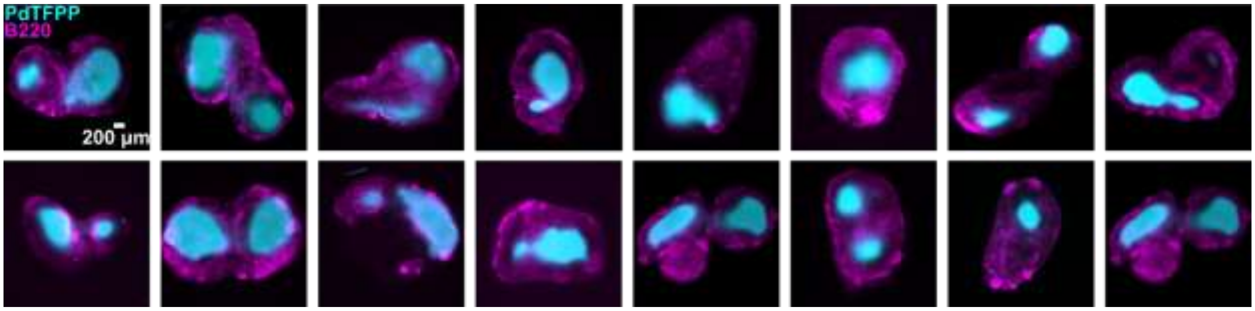

Figure S2: Distribution of oxygen consumption in lymph node slices from naïve mice. Each image was collected 5 minutes after the lymph node tissue slice was placed onto the perfusion chamber and the flow of oxygenated PBS was stopped. Oxygen was consumed mostly in T cell regions of the slices (PdTFPP oxygen sensor in cyan). The B cell regions were stained with B220 (magenta).

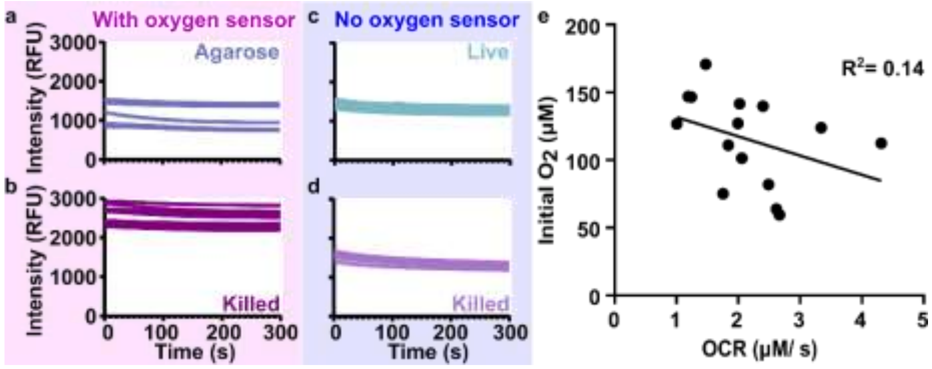

Figure S3: The photobleaching rates of PdTFPP oxygen sensor film and of tissue autofluorescence in oxygenated PBS. (a,b) Plot of fluorescence signal from the PdTFPP oxygen sensor over time during imaging of (a) an agarose slice or (b) an ethanol-killed tissue slice. The shutter was closed between the takes. (c,d) Plots of fluorescence signal from (c) live and (d) ethanol-killed slices without the oxygen sensor over time. N = 6 slices for all conditions; each curve shows one slice. Same y-axis scale as in (a,b). (e) Correlation plot between the initial O_2 concentration and the initial rate of O_2 consumption in live tissue slices, for the data shown in Figure 4e. The strength of a linear association between the two variables was measured using a two-tailed Pearson correlation coefficient test, and the R squared value of 0.14 indicated no correlation between the variables.

Captions for supplemental movies

Movies showing fluorescent signal from PDTFPP oxygen sensor overlaid with live and killed lymph node tissue slices. Images were taken every 5 seconds for a total of 5 minutes. PdTFPP signal is in cyan color and B220 (B cell biomarker) is in magenta color. Three movies are provided; two representative of live slices and one for killed slices.

Supplemental Tables

Table S1: Fluorescent antibodies used to label cells for fluorescence microscopy.

Biomarker/ reagents	Clone	LOT	CAT	Vendor
CD16/32	93	B298973	101302	Biolegend
B220	RA3-6B2	B243962	103226	Biolegend

Supplemental assumptions and calculations

Dissolved oxygen calculations and assumptions: Converting mmHg to mM O_2

Dissolved oxygen concentration in solution is affected by factors such as atmospheric pressure and the solubility of $O_{2(g)}$ in the liquid, which is inversely proportional to solvent temperature and ionic strength. To calculate the solubility of oxygen gas in PBS, we used Henry's law of solubility (Eq. S 1). This Law provides a mathematical description of gas solubility in a liquid medium. According to Henry's law, at a constant temperature, the solubility of a gas in a liquid is directly proportional to the partial pressure of the gas above the liquid at equilibrium.

$$C = \partial P_{gas} / H \qquad \text{Eq. S 1}$$

Where C is the solubility of a gas at a fixed temperature in a particular solvent (mM), H is Henry's law constant (mmHg/ mM), and ∂P_{gas} is the partial pressure of the gas (mmHg). The Henry's law constant for a solution is dependent on the concentration of electrolytes and proteins, atmospheric pressure, and temperature of the solution. Here, we assumed a standard atmospheric pressure of 760 mmHg and temperature of 37 °C for all experiments. Ionic strength of the 1x PBS used here (LONZA; Catalog No: 17-516F) was calculated to be 166 mM at pH 7.4.

Next, we determined the partial pressure of O_2 in the cell culture incubator and in the stage-top chamber used for the optical assay. At 37 °C and 100% humidity (inside incubator), water vapor exerts a partial pressure of 47 mmHg.[1] Therefore, water vapor will make up 6.2% of the total gas at sea level (47 mmHg/ 760 mmHg). To calculate the final O_2% in the gas phase under different conditions used for cell culture, we used the following equation:

$$\%O_2\,final = (\%O_2\,initial \times (1 - \%gas_{(1)} - \%gas_{(n)} + \ldots)) \times 100 \qquad \text{Eq. S 2}$$

Therefore, for the following conditions we have the following sample calculation:

- Humidified incubator without 5% CO2 (Place et al. 2017)

  $\%O_2$ final = [%O2 initial$_{(atmospheric)}$ × (1- % H_2O vapor)] × 100

  $\%O_2$ final = [0.21 × (1 – 0.062)] × 100 = 19.7%

  PO_2 = 760 mmHg × $\%O_2$

  PO_2 = 760 mmHg × 0.197 = 149.7 mmHg

The extrapolated dissolved oxygen concentration at ionic strength of 166 mM in a humidified incubator without CO_2 is 0.195 mM (Place et al. 2017, Supplemental Table 1).[2–4] Henry's constant under these known conditions can be solved as follows:

- Incubator without 5% CO_2

  $H = \partial P_{gas}/C$ = 149.7 mmHg / 0.195 mM; H = 767.69 mmHg/mM

Therefore, we used 767.69 mmHg/mM as the Henry's constant for conversions between mmHg to mM O_2 throughout the manuscript.

Shear stress calculation for flow of PBS in perfusion chamber

To estimate the fluid shear stress (*FSS*) resulting from the flow of 1x PBS through the perfusion chamber, we assumed a thin rectangular cross section (Eq. S 3)[5]:

$$FSS = (6\eta Q) / (h^2 \times w) \qquad \text{Eq. S 3}$$

Where η is fluid viscosity, Q is fluid flow rate, h is height of the bath, and w is width of the bath. For the closed bath chamber, we have Q = 3.6 mL/min = 0.06 cm^3/s, η of PBS = approximately 1.00×10^{-3} Pa·s,[6] h = 0.25 cm, and w = 1.3 cm (Warner Instruments). Thus, the fluid shear stress is calculated as

$$FSS = \frac{[(6)(1.00 \times 10^{-3}\ Pa.s)\left(0.06\frac{cm^3}{s}\right)]}{(0.25\ cm)^2\ (1.3\ cm)} = 0.004\ Pa$$

Converting from Pa to dyn/cm^2 (1 Pa = 10 dyn/cm^2), we have an estimated *FSS* of 0.044 dyn/cm^2 in the chamber under these conditions.

Rates of oxygen consumption: Assumptions and calculations

At each time point, the fluorescence intensities from the oxygen sensor film were collected in mean grey value, which was converted to [dissolved O_2] (mM) by using the oxygen calibration curve for the perfusion chamber (Stern-Volmer equation, Eq. 1 from main text) and Henry's law. After generating a plot of $[O_2]$ (mM) vs time (s), the initial portion of the curve was fit with a linear fit, and the mean rate of consumption per tissue slice (mM/s) was obtained from the absolute value of the slope (Figure 4d-f).

To compare the optical assay to the results from a Seahorse assay, it was necessary to convert between units of mM/s per T cell zone and pmol/min/cell. Doing so required a series of assumptions and simplifications, yielding an order-of-magnitude comparison between the two measurements. To convert from molarity to moles by using the molarity equation (Eq. S 4),

$$\textit{Molarity = moles of substrate / volume of solution} \qquad \text{Eq. S 4}$$

We estimated the average volume (area x height) of the T cell zone in a lymph node slice. To determine an average area, we performed immunofluorescence labelling of lymph node slices, defined the T cell zones as central B220-negative regions (see e.g. Figure 3c from main text), and measured the areas of these

regions using ImageJ. Measurements in 12 naïve lymph node slices from 6 wks old male and female mice yield an average area of $1.01 \times 10^6 \pm 0.26 \times 10^6$ μm^2. Assuming a thickness of 300 μm, this yielded an average volume of 3.02×10^8 μm^3.

To convert from moles per T cell zone to moles per cell, we estimated an average number of T cells per T cell zone. To do so, we referred to the average percent composition of CD3+ T cells per lymph node (50 ± 17%) and the average number of cells per 300-μm-thick lymph node slice ($(0.56 \pm 0.16) \times 10^6$ cells).[7] Assuming each murine lymph node slice had two T cell zones (most have one or two zones; see Figure S2), this yielded a rough order-of-magnitude estimate of 140,000 T cells per T cell zone in a lymph node slice.

As an example, using these numbers, for a T cell zone in which the oxygen consumption rate was 19×10^{-4} mM/sec as measured by the optical assay, we estimated that 2.4×10^{-4} pmol/min/cell was consumed.

Supporting references

(1) Pittman, R. N. Regulation of Tissue Oxygenation. *Colloq. Ser. Integr. Syst. Physiol. Mol. Funct.* **2011**, *3* (3), 1–100. https://doi.org/10.4199/C00029ED1V01Y201103ISP017.

(2) Carpenter, J. H. New Measurements of Oxygen Solubility in Pure and Natural Water1. *Limnol. Oceanogr.* **1966**, *11* (2), 264–277. https://doi.org/10.4319/lo.1966.11.2.0264.

(3) Koppenol, W. H.; Butler, J. Energetics of Interconversion Reactions of Oxyradicals. *Adv. Free Radic. Biol. Med.* **1985**, *1* (1), 91–131. https://doi.org/10.1016/8755-9668(85)90005-5.

(4) Place, T. L.; Domann, F. E.; Case, A. J. Limitations of Oxygen Delivery to Cells in Culture: An Underappreciated Problem in Basic and Translational Research. *Free Radic. Biol. Med.* **2017**, *113*, 311–322. https://doi.org/10.1016/j.freeradbiomed.2017.10.003.

(5) Oh, K. W.; Lee, K.; Ahn, B.; Furlani, E. P. Design of Pressure-Driven Microfluidic Networks Using Electric Circuit Analogy. *Lab. Chip* **2012**, *12* (3), 515–545. https://doi.org/10.1039/C2LC20799K.

(6) Jun Kang, Y.; Yeom, E.; Lee, S.-J. A Microfluidic Device for Simultaneous Measurement of Viscosity and Flow Rate of Blood in a Complex Fluidic Network. *Biomicrofluidics* **2013**, *7* (5), 054111. https://doi.org/10.1063/1.4823586.

(7) Belanger, M. C.; Ball, A. G.; Catterton, M. A.; Kinman, A. W. L.; Anbaei, P.; Groff, B. D.; Melchor, S. J.; Lukens, J. R.; Ross, A. E.; Pompano, R. R. Acute Lymph Node Slices Are a Functional Model System to Study Immunity Ex Vivo. *ACS Pharmacol. Transl. Sci.* **2021**, *4* (1), 128–142. https://doi.org/10.1021/acsptsci.0c00143.
